# Prevalence of pityriasis rosea in the United States: A cross-sectional study using the *All of Us* database

**DOI:** 10.1016/j.jdin.2022.04.006

**Published:** 2022-05-19

**Authors:** Tejas P. Joshi, Gianmarco A. Calderara, Jules B. Lipoff

**Affiliations:** a School of Medicine, Baylor College of Medicine, Houston, Texas; b Department of Dermatology, Perelman School of Medicine, Philadelphia, Pennsylvania; c Leonard Davis Institute of Health Economics, University of Pennsylvania, Philadelphia, Pennsylvania

**Keywords:** diversity, ethnic skin, pityriasis rosea, prevalence, representation, skin of color

*To the Editor:* Pityriasis rosea (PR) is a common papulosquamous disease; however, its epidemiology is not well established. Single-center studies, conducted mostly outside the United States, have estimated the prevalence of PR to be 0.39-4.80%^1^; however, there are no published estimates of PR prevalence from a diverse, nationwide cohort of American patients. We aimed to estimate the prevalence of PR using the *All of Us* database, a recently launched initiative by the National Institutes of Health that strives to include communities that have been historically underrepresented in research (eg, gender, racial, and sexual minorities and rural populations).^2^

This study was deemed IRB exempt by the University of Pennsylvania. We performed a cross-sectional analysis of the *All of Us* database by identifying patients with a diagnosis of PR using ICD-9-CM code 696.3 and ICD-10-CM code L42. Electronic medical records of each patient with PR were then analyzed to collect data on age, sex, and self-identified race. We utilized the Wald method with 95% confidence intervals (CI) to calculate the overall prevalence of PR.

Currently, the *All of Us* database has enrolled 327,654 participants. We identified 687 patients with PR, representing an overall prevalence of 0.21% (95% CI, 0.19-0.23) (Table I). The average age at diagnosis was 36.5 years (standard deviation 16.1). The prevalence was highest in the 18-25 age group (0.77%, 95% CI 0.63-0.92), decreasing with increasing age. We observed a female-to-male predominance of 3:1. Prevalence in specific racial groups included 0.21% (95% CI, 0.17-0.25) in Hispanic, 0.17% (95% CI, 0.09-0.25) in Asian, 0.24% (95% CI, 0.20-0.28) in Black, and 0.20% in white patients (0.20%, 95% CI, 0.18-0.22).

**Table I:** Prevalence of pityriasis rosea in the United States, stratified by age

Group	Total population, *N*	PR cases, *N*	Prevalence, % (95% CI)	Female, *N* (%)	Age (mean, SD)
Overall	327,654	687	0.21 (0.19-0.23)	514 (74.8)	36.5, 16.1
White	177,648	353	0.20 (0.18-0.22)	245 (69.4)	36.5, 16.1
Black	69,087	166	0.24 (0.20-0.28)	135 (81.3)	36.0, 14.6
Hispanic	60,540	128	0.21 (0.17-0.25)	104 (81.3)	34.8, 14.3
Asian	11,040	19	0.17 (0.09-0.25)	14 (73.7)	38.0, 10.2
Age					
18-25	14,342	111	0.77 (0.63-0.92)	89 (80.2)	22.0, 2.16
26-30	22,801	92	0.40 (0.32-0.49)	74 (80.4)	28.1, 1.46
31-35	25,669	77	0.30 (0.23-0.37)	58 (75.3)	32.9, 1.44
36-40	25,741	74	0.29 (0.22-0.35)	57 (77.0)	38.1, 1.39
41-50	48,030	115	0.24 (0.20-0.28)	88 (76.5)	45.4, 2.92
51-65	99,523	114	0.11 (0.09-0.14)	77 (67.5)	57.3, 4.03
65+	91,548	28	0.03 (0.02-0.04)	14 (50.0)	72.4, 5.20

*CI*, Confidence interval; *PR*, pityriasis rosea; *SD*, standard deviation.

Given similar prevalence among all ethnicities, we might expect educational materials to depict PR equally in all skin types. However, recent analysis of educational resources has found PR less well represented in dark skin compared to light skin.^3^ This trend is especially concerning given that the presentation of PR in lighter skin types (pink scaly plaques) may be morphologically different from that in darker skin types (gray/violaceous plaques, often in an inverse pattern),^4^ highlighting the importance of diverse educational representation.

Our analysis has limitations. As the *All of Us* database is organized by billing codes, we likely missed cases of PR simply coded as “rash”; thereby, our prevalence calculation likely represents an underestimate of true PR prevalence. In addition, our data set did not include patients younger than 18 years of age. Furthermore, the *All of Us* database is 54% white, 21% Black, and 18.5% Hispanic, while the United States population is currently 60% white, 13.4% Black, and 18.5% Hispanic.^5^ Thus, our prevalence calculation of PR among Black Americans may be an overestimate because of the increased representation of Black Americans in *All of Us*.

Altogether, our data suggest PR is a common dermatosis prevalent across all racial groups. Given the prevalence observed in Black patients, we advocate for greater educational representation of PR in darker skin types. Further epidemiologic studies that are not restricted by billing codes and include pediatric patients may validate our findings.

## Conflicts of interest

None disclosed.
